# Associations of Multiparametric Breast MRI Features, Tumor-Infiltrating Lymphocytes, and Immune Gene Signature Scores Following a Single Dose of Trastuzumab in HER2-Positive Early-Stage Breast Cancer

**DOI:** 10.3390/cancers15174337

**Published:** 2023-08-30

**Authors:** Laura C. Kennedy, Anum S. Kazerouni, Bonny Chau, Debosmita Biswas, Rebeca Alvarez, Grace Durenberger, Suzanne M. Dintzis, Sasha E. Stanton, Savannah C. Partridge, Vijayakrishna Gadi

**Affiliations:** 1 Department of Medicine, Vanderbilt University Medical Center, Nashville, TN 37232, USA; 2 Department of Medicine, University of Washington, Seattle, WA 98195, USA; 3 Fred Hutchinson Cancer Center, Seattle, WA 98109, USA; 4 Department of Radiology, University of Washington, Seattle, WA 98195, USA; 5 Department of Pathology, University of Washington, Seattle, WA 98195, USA; 6 Cancer Immunoprevention Laboratory, Earle A. Chiles Research Institute, Portland, OR 97213, USA; 7 Department of Medicine, University of Illinois Chicago, Chicago, IL 60612, USA; 8 Translational Oncology Program, University of Illinois Cancer Center, Chicago, IL 60612, USA

**Keywords:** Breast MRI, trastuzumab, immune biomarker, breast cancer, tumor-infiltrating lymphocytes

## Abstract

**Simple Summary:**

Imaging biomarkers that permit non-invasive, real-time monitoring of the tumor microenvironment could serve a critical role to facilitate treatment personalization, particularly in the context of new immunotherapies. The aim of this prospective pilot study was to investigate the value of breast magnetic resonance imaging (MRI) features as early markers of treatment-induced immune response after a single dose of trastuzumab in early HER2+ breast cancer. Our findings showed measures of change in peak percent enhancement on dynamic contrast-enhanced MRI and pre-treatment apparent diffusion coefficient on diffusion-weighted MRI to correlate with immune response as defined by tumor-infiltrating lymphocytes and immune-active gene signature scores. MRI measures hold potential to serve as biomarkers of tumor microenvironment alterations to guide treatment decisions in early breast cancer.

**Abstract:**

Dynamic biomarkers that permit the real-time monitoring of the tumor microenvironment response to therapy are an unmet need in breast cancer. Breast magnetic resonance imaging (MRI) has demonstrated value as a predictor of pathologic complete response and may reflect immune cell changes in the tumor microenvironment. The purpose of this pilot study was to investigate the value of breast MRI features as early markers of treatment-induced immune response. Fourteen patients with early HER2+ breast cancer were enrolled in a window-of-opportunity study where a single dose of trastuzumab was administered and both tissue and MRIs were obtained at the pre- and post-treatment stages. Functional diffusion-weighted and dynamic contrast-enhanced MRI tumor measures were compared with tumor-infiltrating lymphocytes (TILs) and RNA immune signature scores. Both the pre-treatment apparent diffusion coefficient (ADC) and the change in peak percent enhancement (DPE) were associated with increased tumor-infiltrating lymphocytes with trastuzumab therapy (r = −0.67 and -0.69, *p* < 0.01 and *p* < 0.01, respectively). Low pre-treatment ADC and a greater decrease in PE in response to treatment were also associated with immune-activated tumor microenvironments as defined by RNA immune signatures. Breast MRI features hold promise as biomarkers of early immune response to treatment in HER2+ breast cancer.

## 1. Introduction

The widespread adoption of immunotherapy in a variety of solid tumor types has created a need for methods to both interrogate the immune status of the tumor microenvironment at baseline and to monitor for treatment-induced immune response. This is particularly true in early breast cancer, where immune-based treatments are employed in both human epidermal growth factor receptor 2 (HER2)-amplified and triple-negative breast cancers. Incorporating biomarkers that can provide serial assessment of the tumor immune response into clinical care can promote the goals of achieving pathologic complete responses (pCR) while minimizing unnecessary treatments and toxicities.

Treatment selection in early breast cancer has historically been guided by immunohistochemistry (IHC) for the prognostic markers estrogen receptor (ER), progesterone receptor (PR), and HER2 [1,2]. However, immunohistochemistry and other tissue-based biomarkers are innately disadvantaged as strategies for monitoring dynamic changes in the tumor microenvironment. Imaging-based biomarkers offer the potential to monitor the entire tumor volume over time with minimal risk to the patient, enabling early therapeutic adjustments to optimize cancer outcomes.

In HER2+ breast cancer, trastuzumab, a humanized mouse monoclonal antibody against the human HER2 receptor, is an essential component of treating the HER2-positive breast cancer subtype [3] and is known to stimulate an anti-tumor immune response [4,5,6,7]. Patients with increasing levels of tumor-infiltrating lymphocytes (TILs) in response to trastuzumab have better survival [4,8]. Both MRI and PET have shown promise as potential predictors of pre-surgical treatment response in the context of HER2+ breast cancer [9,10]. In this report, we focus on breast MRI, which is part of the standard pre-treatment evaluation at many cancer centers. There is evidence that breast MRI parameters such as functional tumor volume (FTV) and apparent diffusion coefficient (ADC) can be used as early predictive markers of chemotherapy response in local breast cancer [9,11,12]. Recent work also has shown that these breast MRI parameters may reflect the TIL composition of the tumor [13,14,15].

Herein, we present the results of a pilot window study in fourteen patients with early stage HER2+ breast cancer who underwent a pre- and post-treatment MRI after receiving a single dose of trastuzumab. The primary aims were to assess the magnitude and variability of early immune response (approximately two weeks after treatment) and investigate the ability of breast MRI features to reflect the level of immune response. Pre- and post-treatment tissue was collected for immune microenvironment assessment with TILs and RNA-based immune signatures and then correlated with MRI parameters. Our study found that breast MRI has potential as a monitoring strategy for the tumor immune response to trastuzumab immunotherapy in early HER2+ breast cancer.

## 2. Materials and Methods

### 2.1. Patient Criteria and Enrollment

The study was conducted in accordance with the Declaration of Helsinki, and approved by the Institutional Review Board of the Fred Hutchinson Cancer Center (Protocols IR#6687, approved June 2012, and IR#7889, approved February 2013). Subjects were enrolled at the University of Washington and Fred Hutchinson Cancer Center (formerly the Seattle Cancer Care Alliance) between January 2013 and September 2019 (ClinicalTrials.gov #NCT03738553). Patients with a new diagnosis of HER2+ breast cancer (IHC 3+ or 2+ amplified by in situ hybridization), a minimum size of 1 cm, and a plan for curative-intent therapy including surgery were eligible. Patients with breast tumors that were poorly visualized on the pre-treatment MRI, could not complete a breast MRI, or were pregnant and/or breastfeeding were excluded. Informed consent was obtained prior to initiating study procedures.

**Figure 1:**
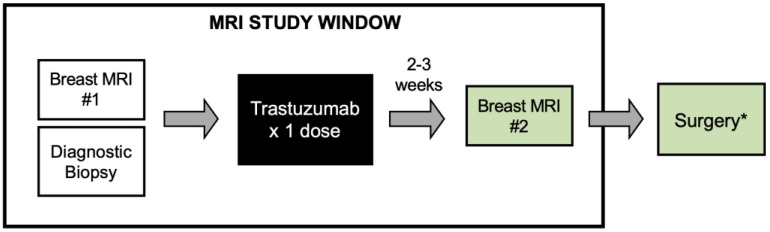
Study schema. Patients with newly diagnosed and untreated HER2+ breast cancer were eligible for the study. Patients enrolled in the study underwent two breast MRI exams, before and after receiving a single dose of trastuzumab. Tissue was obtained pre-treatment from the diagnostic biopsy and post-treatment tissue was typically obtained at time of surgery. * For patients planning to undergo a full course of neoadjuvant treatment, a research biopsy was carried out prior to initiating chemotherapy.

Patients were imaged with breast MRI prior to surgery and prior to receiving a loading dose (8 mg/kg) of trastuzumab and had repeat imaging with breast MRI approximately 2–3 weeks after treatment. Pre-treatment tissue samples were obtained from each patient’s archival diagnostic biopsy. Post-treatment tissue was obtained 2–4 weeks after trastuzumab treatment from the patient’s surgical specimen (Figure 1). Patients planning to undergo a full course of neoadjuvant chemotherapy who wished to participate in this study received the run-in dose of trastuzumab prior to initiating neoadjuvant treatment. For those patients, the post-run-in tissue was obtained with a biopsy prior to the patient starting neoadjuvant chemotherapy. If desired, patients were permitted to receive pertuzumab with the run-in trastuzumab as per their treatment provider.

### 2.2. Breast MRI Acquisition

All scans were performed at the Fred Hutchinson Cancer Center on a 3 Tesla Philips Achieva Tx system (Philips Healthcare, Best, The Netherlands) with a 16-channel breast coil (Mammotrak, Philips Healthcare). All MRI protocols followed guidelines established by the American College of Radiology breast MRI accreditation program [16,17,18] and included dynamic contrast-enhanced (DCE) MRI and diffusion-weighted (DW) MRI sequences. All scans were acquired in the axial orientation. DCE-MRI was performed using a fat-suppressed *T*_1_-weighted 3D fast gradient echo sequence (enhanced *T*_1_-weighted High Resolution Isotropic Volume Excitation; eTHRIVE) with TR 4.3–6.2 ms, TE 2.2–3 ms, flip angle 10°, in-plane spatial resolution 0.5–0.8 mm, slice thickness 1.3–1.6 mm, and scan duration 2–3 mins. One pre- and at least two sequential post-contrast sequences were acquired, with k-space centered at approximately 120 s (initial phase) and 360–480 s (delayed phase) after contrast injection. The contrast agent administered was 0.1 mmol/kg body weight gadoteridol (ProHance, Bracco Diagnostics, Milan, Italy) at a rate of 2 cc/s followed by a 15–20 cc saline flush. DW-MRI was acquired using a diffusion-weighted single shot echo-planar imaging sequence with parallel imaging and fat suppression (spectral attenuated inversion recovery; SPAIR), TR 3500–5336 ms, TE 61–66 ms, SENSE reduction factor 3, in-plane resolution 1.5–1.8 mm, slice thickness 4 mm, number of slices 30, and multiple applied b-values including 0, 100, and 800 s/mm^2^, with scan duration of approximately 4:30 mins.

### 2.3. Breast MRI Analysis

All MR image analyses were performed with observers blinded to pathologic outcomes, using customized software tools for DCE- and DW-MRI measurements as detailed below. Tumor segmentation and quantification were performed by researchers (B.C., D.B., and A.K.) under guidance of an imaging scientist (S.C.P. with over 15 years of experience in quantitative breast MRI) who reviewed all tumor region-of-interest (ROI) segmentations.

### 2.4. DCE-MRI Kinetic Analysis 

DCE-MRI volumes were first co-registered using a commercially available computer-aided evaluation software tool (CADstream, Merge Healthcare Inc., Chicago, IL, USA) to correct for any misregistration due to patient motion. Next, volumetric kinetics analysis was performed using in-house software written in ImageJ (NIH, public domain, Bethesda, MD, USA), as previously described [19,20]. Contrast kinetics were characterized by several parameters that reflect the delivery of contrast; [initial peak percent enhancement (peak PE)], clearance of contrast [peak delayed signal enhancement ratio (peak SER) and washout fraction (WF)], and functional tumor volume (FTV). Initial PE (units, %) reflects the degree of signal enhancement in the tumor at 120 s after contrast delivery, calculated by

(1) $$PE=\frac{S_{1}-S_{0}}{S_{0}}\times 100$$

where *S*_0_ is the MRI signal intensity prior to contrast and *S*_1_ is the MRI signal intensity 120 s after contrast delivery. SER is a unitless index that reflects the rate of contrast washout in the tumor between initial and delayed phase post-contrast imaging, calculated by:

(2) $$SER=\frac{S_{1}-S_{0}}{S_{2}-S_{0}}$$

where *S*_2_ is the MRI signal intensity at 360 to 480 after contrast delivery. PE and SER were calculated for each voxel within the tumor ROIs, with SER calculated only for voxels with PE ≥ 50%. FTV (units, cm^3^) was calculated by summing volumes for all voxels with PE ≥ 50%. Additionally, WF percentage was calculated as the fraction of all tumor voxels exhibiting washout (defined as those with PE ≥ 50% and SER ≥ 1.1). The software automatically identified independent hot-spot (‘peak’) regions of initial PE and SER that were defined as 3 × 3 × 3 voxel regions producing the highest mean initial PE and SER value, respectively. FTV, PE, SER, and WF were determined for MRI #1 and MRI #2. DFTV, DPE, DSER, and DWF were determined by subtracting the pre-treatment value from the post-treatment value.

### 2.5. DW-MRI Analysis

DW-MRI was analyzed with custom software developed in Matlab (Mathworks, Natick, MA), as previously described [21]. Briefly, ADC maps (units mm^2^/s) were calculated using a linear least-squares fit of the log of the signal intensities at *b*-values (0, 100, and 800 s/mm^2^) and a classic monoexponential decay model:

(3) $$S\left(b\right)=S_{0}\times e^{-b\times ADC}$$

where *S*(*b*) is the signal intensity with diffusion weighting *b* and *S*_0_ is the signal with no diffusion weighting. DCE-MRI and *T_2_*-weighted images were referenced to avoid areas of cyst and necrosis and to restrict measures to viable solid tumor regions. Mean ADC values were calculated across lesion ROI voxels for both MRI #1 and MRI #2. DADC was calculated by subtracting the pre-treatment ADC from the post-treatment ADC.

### 2.6. Tumor Microenvironment Evaluation

#### 2.6.1. TIL Assessment

Two separate reads for stromal TIL measurement were performed independently by two trained clinical breast pathologists on hematoxylin and eosin (H&E)-stained slides from the pre- and post-treatment tissue specimens following the International TILs Working Group guidelines [22]. TILs were classified into deciles for analysis and the two decile reads were averaged together to establish the final decile for analysis. The change in TILs (DTIL) was determined by subtracting the post-trastuzumab TILs from the pre-trastuzumab TILs. Immune response was defined as an increase of greater than one decile in TILs between the pre- and post-treatment timepoints, and non-responders had an increase of one decile or less between pre- and post-treatment timepoints. 

#### 2.6.2. RNA Isolation

Unstained formalin-fixed and paraffin-embedded slides were deparaffinized using Citrisolv, washed using 100% ethanol followed by nuclease-free water and allowed to air dry. The dried tissue section was coated with 3% glycerol and scraped off the slide into a polymerase chain reaction (PCR) tube. The total RNA was then extracted using the Qiagen AllPrep DNA/RNA formalin-fixed paraffin-embedded (FFPE) kit (Qiagen, Germantown, MD, USA).

#### 2.6.3. Immune Signature Scores

Immune signature scores were obtained through a commercially available service (Nanostring, Seattle, WA, USA) using the Nanostring PanCancer IO360 panel as previously described [23]. Briefly, samples were submitted to Nanostring for gene expression profiling using the nCounter analysis system. Samples that failed quality control metrics were excluded from the analysis. Raw data were analyzed using nSolver Analysis Software through the IO 360 Data Analysis Service (Nanostring, Seattle, WA, USA). Differential gene expression and hierarchical clustering were performed as previously described [23].

### 2.7. Statistical Analysis

#### 2.7.1. Correlation of MRI Features with TILs

The primary objectives of this prospective pilot study were to (1) assess the magnitude and variability of immune response two weeks after a single dose of targeted anti-HER2 therapy and (2) to investigate the ability of DCE-MRI and DW-MRI to reflect an increased level of immune response based on the number of TILs. The statistical analysis plan was exploratory with a target accrual of 12–14 patients. Pre- and post-treatment stromal TIL levels for the cohort were compared using a paired *t*-test. Within immune response groups defined by a change in TILs, pre- and post-treatment MRI features were compared using a paired *t*-test. Comparison of MRI features with TILs levels was performed using Spearman’s rank order correlation. Correction for multiple comparisons was not performed; *p* < 0.05 indicated significance. 

#### 2.7.2. Correlation of MRI Features with Tumor Microenvironment Gene Expression

Data analysis was performed using the Nanostring Data Analysis service and the PanCancer IO 360 Biological Signatures were generated (https://nanostring.com/products/ncounter-assays-panels/oncology/pancancer-io-360/, accessed on 16 August 2023); a description of the methodology is available in the Nanostring online documentation, summarized briefly here. For purposes of comparison, genes were normalized using the ratio of the expression value to the geometric mean of all housekeeping genes on the panel. The housekeeper-normalized data were Log (2) transformed. Signatures were adjusted with constants to express values in a similar range. Differential expression was fit on a per-gene or per-signature basis using a linear model for analyses without a blocking factor. The statistical model uses the expression value or signature score as the dependent variable and fits a grouping variable as a fixed effect to test for differences in the levels of that grouping variable:

Expression(gene or signature) = μ + Group + ε(4)

*p*-values were adjusted within each analysis, gene or signature, and with the grouping variable level difference *t*-test using the Benjamini and Yekutieli False Discovery Rate (FDR) adjustment to account for correlations amongst the tests [24]. All models were fit using the limma package in R. Heatmaps were generated using hierarchical clustering and were annotated for timepoint (pre- or post-treatment), hormone receptor status, significant imaging features (identified to correlate with TIL response), and the presence or lack of a tumor-infiltrating lymphocyte (TIL) response (response was defined as an increase greater than 1 decile in TILs between the pre- and post-treatment timepoints).

## 3. Results

### 3.1. Patient Demographics

Fourteen women (median age 52 years, range 37–69 years, Table 1), all diagnosed with invasive ductal carcinoma, were enrolled in the study between January 2013 and September 2019. Three of the patients had biopsy-proven lymph node involvement at time of diagnosis; these patients proceeded with a full course of neoadjuvant chemo- and HER2-directed therapy after the study window was complete (following the run-in dose of trastuzumab). Half of the patients were estrogen-receptor-positive (i.e., Allred score 3/8 or greater).

The median time from administration of the run-in trastuzumab dose to the post-treatment MRI was 14 days (range 11–36 days). All 14 patients had pre- and post-treatment breast MRIs as per the study schema (Figure 1) and pre-treatment tissue available. One patient did not have post-treatment tissue available for analysis because there was no research biopsy after the single dose of trastuzumab.

### 3.2. Peak PE and WF Correlate with an Immune Response Post-Trastuzumab Administration

**Figure 2:**
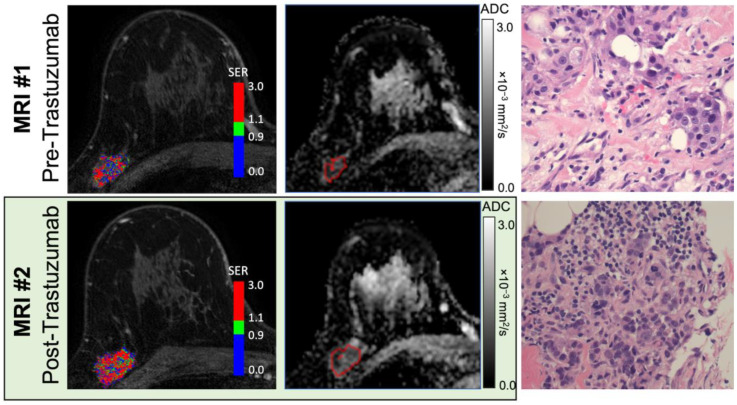
Paired pre- and post-treatment MRI and histology assessments in a 51-year-old woman with Grade 3 ER+/HER2+ invasive ductal carcinoma (Patient ID #13). Shown are representative signal enhancement ratio maps generated from dynamic contrast-enhanced MRI (**left**), apparent diffusion coefficient maps from diffusion-weighted MRI (middle; tumor region-of-interest is shown), and hematoxylin and eosin (H&E)-stained tissue specimens at ×400 magnification (**right**) obtained before and after a run-in treatment of trastuzumab (top and bottom row, respectively). Prior to treatment, quantitative MRI analysis measured functional tumor volume (fTV) of 2.1 cc, washout fraction 35%, initial peak percent enhancement (PE) 207%, peak signal enhancement ratio (SER) 1.87, and apparent diffusion coefficient (ADC) 0.61 × 10^−3^ mm^2^/s. MRI obtained 14 days after treatment demonstrated increases in both vascularity- and cellularity-related metrics (fTV 5.0 cc, washout fraction 47%, PE 231%, SER 2.17, and ADC 0.66 × 10^−3^ mm^2^/s). Pathologist assessment of stromal tumor-infiltrating lymphocyte (TIL) level identified an increase from pre- (≤9%) to post-treatment (10–19%), reflecting a treatment-induced immune response.

**Figure 3:**
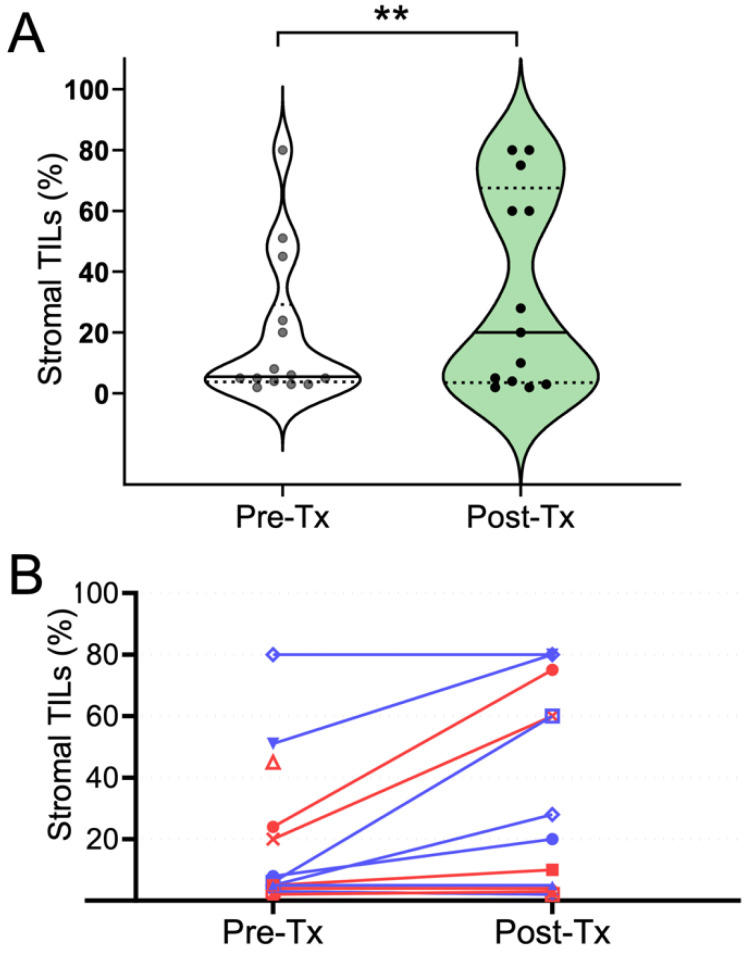
Tumor-infiltrating lymphocyte (TIL) distributions before and after trastuzumab treatment. (**A**). Treatment with trastuzumab generally induced an increase in TILs in the post-treatment specimen compared to the pre-treatment specimen (paired *t*-test, *p* < 0.01). (**B**). Pre-treatment and post-treatment TIL level pairs for individual patients (each patient represented by a unique symbol). Red indicates ER-positive patients, blue indicates ER-negative patients. One ER+ patient had only pre-treatment tissue available **.

To investigate whether imaging features were associated with treatment-induced immune response, pre- and post-trastuzumab MRIs were collected and analyzed. For this study, immune response was defined by TILs. A representative set of MRI data and histology are shown in Figure 2; individual patient data are available in Table S1. The percentage of stromal TILs increased (defined as an increase greater than 1 decile) after treatment with trastuzumab in 6 out of 13 patients, with the median TILs going from 6% to 20% in the cohort (*p* < 0.01, Figure 3A). When stratified by hormone-receptor status, four out of seven estrogen receptor (ER)-negative patients had an increase in TILs with trastuzumab treatment and two out of the five evaluable ER-positive patients had an increase in TILs (Figure 3B and Figure S1). Two ER-negative and one ER-positive patient had TIL-enriched (TILs ≥ 30%) tumors at baseline.

Among the DCE-MRI features, both peak PE and WF were significantly association with ΔTIL. DPE exhibited a strong negative correlation with DTILs (*r* = −0.69, *p* < 0.01), and a moderate negative correlation with both the pre- and post-treatment TILs (*r* = −0.45 and −0.51, respectively, *p* < 0.05, Table 2). Both pre-treatment WF and pre-treatment PE exhibited moderate positive correlations with DTILs (*r* = 0.51 and 0.48, respectively, *p* < 0.05, Table 2). When grouped together, tumors with an immune response had a significant decrease in peak PE post-treatment (*p* = 0.02, paired *t*-test, Figure S2A), while peak PE did not change significantly in tumors without an immune response. No other DCE-MRI features demonstrated significant differences between pre- and post-treatment when separated by immune response (Figure S2B–E).

From DW-MRI, pre-treatment ADC exhibited a moderate negative correlation with the post-treatment TIL percentage (*r* = −0.51, *p* < 0.05) and a moderate to strong negative correlation with DTILs (*r* = −0.67, *p* < 0.01, Table 2). Post-treatment ADC exhibited a moderate negative correlation with the DTILs (*r* = −0.49, *p* < 0.05). When separated by immune response, there were no statistically significant differences in ADC between the pre- and post-treatment results for either group (Figure S2E).

### 3.3. Diminished Peak PE Was Associated with an Immune-Activated Gene Signature 

**Figure 4:**
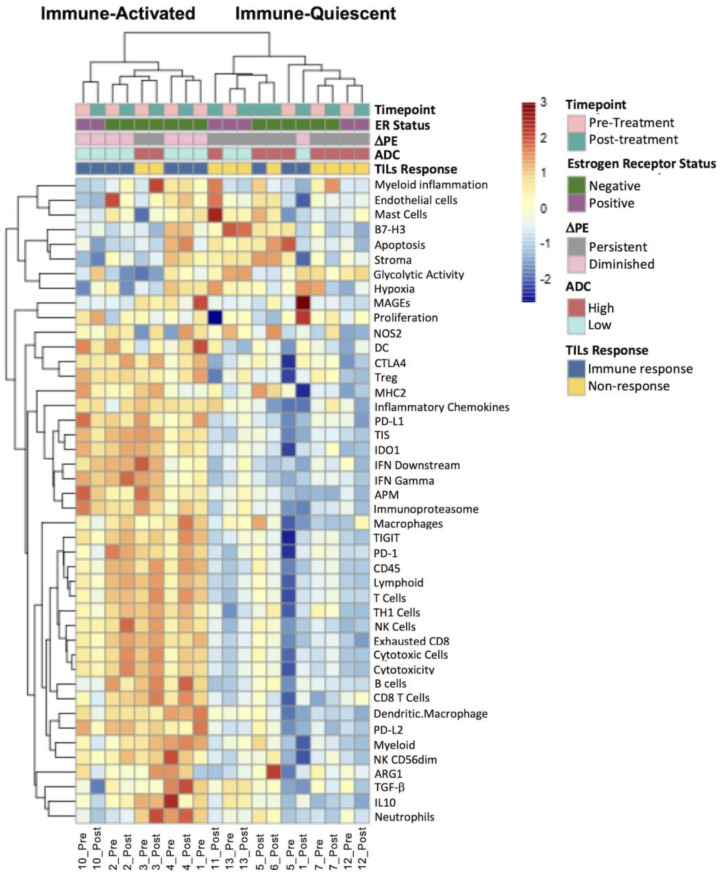
Heatmap of RNA immune signatures for pre- and post-trastuzumab samples. Hierarchical clustering of RNA signatures of pre- and post-trastuzumab tissue show two distinct groups: immune-activated and immune-quiescent. The heatmap shows the relative expression of microenvironment markers, as well as imaging and histology features. For DPE (median, −10%), ‘diminished’ corresponded with a decrease in PE of 10% or more with treatment while ‘persistent’ correlated with either an increase in PE or a less than 10% decrease in response to treatment. For pre-treatment ADC values (median, 0.92 × 10^−3^ mm^2^/s), ADC > 0.92 × 10^−3^ mm^2^/s was considered “high”, and “low” otherwise. Immune-activated tumors showed relatively high expression of dendritic cells, cytotoxic cells, and CD45, whereas immune-quiescent tumors showed relatively low expression of these markers. Immune-activated tumors additionally demonstrated more diminished delta PE, low ADC, and TILs response compared to immune-quiescent tumors.

To further evaluate the alignment of MRI features with the immune tumor microenvironment, we derived immune signatures from the pre- and post-treatment samples using RNA sequencing. For purposes of this exploratory analysis, patients were split into “persistent” and “diminished” DPE and “high” and “low” pre-treatment ADC based on the median values of DPE and ADC of the cohort. After quality control metrics, nine paired patient samples (pre- and post-treatment) and two non-paired samples were available for analysis (Figure 4). Patients with a decrease in peak PE of 10% or more post treatment were labeled as “diminished DPE”, while a less than 10% decrease or increase in peak PE post treatment was labeled as “persistent DPE”. The patient cohort had two distinct gene signature scores: an immune-activated group with high expression of T cell, NK cell, B cell, dendritic cell, and cytotoxic immune signatures and an immune-quiescent group with relatively low expression of the same signatures. Pre- and post-treatment timepoints generally clustered together for individual patients, suggesting that trastuzumab treatment did not alter the baseline state of the tumor microenvironment for most tumors [25]. Five out of six patients with an immune response clustered with the immune-activated group, while four out of five patients without an immune response clustered with the immune-quiescent group. The single patient (patient 3) that did not have an immune response, as defined by a pre- to post-treatment change in TILs, but clustered with the immune-activated group, had a highly immune-infiltrated tumor at baseline (80–89% TILs at the pre-treatment timepoint) and had persistently high TILs after trastuzumab. 

Both diminished DPE and low pre-treatment ADC were associated with immune-activated tumors, while patients with persistent DPE clustered with immune-quiescent tumors (Figure 4). Patient 3 was an exception, as the peak PE had only a very small decrease with treatment and was more consistent with the persistent DPE group. However, the lack of change in PE does correspond with the overall stability of TILs in the tumor, which were at a high level both before and after treatment. Similarly, patients with low ADC according to the pre-treatment MRI were generally associated with the immune-activated group by gene expression (Figure 4). There were no significant differences in the differential expression of immune signatures between the low- and high-ADC groups. The only immune [4] signature that demonstrated a significant difference in differential expression when comparing the diminished and persistent DPE groups was the dendritic cell immune signature (*p* < 0.05, adjusted for multiple comparisons). 

## 4. Discussion

Tumor immune infiltration has previously been associated with favorable survival outcomes in early breast cancer [4,26,27] and higher rates of pathologic complete response with neoadjuvant therapy [28,29]. Strategies to visualize and monitor tumor immune re-sponse during active treatment do not currently exist. Here, we present the results of a pi-lot study that suggest breast MRI may have value for monitoring immune response in pa-tients with early-stage HER2+ breast cancer. In this study, DPE and pre-treatment ADC had the strongest correlation with immune response as defined by TILs and immune-active gene signature scores. These results suggest that MRI features could provide valua-ble real-time information on the status of the tumor microenvironment for patients treated with trastuzumab. Moreover, response data can inform escalation (or de-escalation) strat-egies to optimize survival outcomes and mitigate unnecessary toxicities from the intensifi-cation of therapy for individual patients.

Most studies evaluating the utility of breast MRI features have focused on the ability to predict the final pathologic response to pre-surgical therapy [9,11,12,30]. However, MRI may offer the ability to evaluate additional pathologic features and monitor response non-invasively over time. Additional studies have shown that MRI features do have some cor-relation with pathologic features such as tumor vascularity, inflammation, cellularity, and TILs [31,32,33,34,35,36,37,38]. The inclusion of these types of features may augment the ability of MRI to predict neoadjuvant treatment response [13,39,40,41]. These previous studies have mainly focused on the pre-treatment (baseline) imaging, but one advantage of an imaging biomarker is the ability to receive on-treatment evaluations. This is especially important in early HER2+ breast cancers, where the pre-treatment tumor microenvironment is not predictive of response, but the tumor microenvironment after even a single dose of trastuzumab is predictive of a treatment response [29]. In this pilot study, the finding that DPE is a key MRI feature associated with immune response places emphasis on the importance of post-treatment assessment, which is straightforward to obtain with MRI.

In addition, this study illustrates some of the limitations of using tissue-based biomarkers obtained from a single biopsy, which can only reflect a limited portion of the tumor. For example, patient 5 had an immune response as defined by TILs but a tumor microenvironment that was relatively cold as defined by immune signature scores, which are a more comprehensive evaluation. The MR imaging features of persistent DPE and high pre-treatment ADC aligned with a more quiescent immune microenvironment, suggesting that MRI accurately tracked the tumor microenvironment versus the TIL assessment in this instance. 

There are limitations to our study and opportunities for future work. The goal of this study was to assess the potential of MRI features as a minimally invasive biomarker to predict immune response to trastuzumab. This was a proof-of-principle study, and we acknowledge that further studies with larger cohorts and using more advanced methods (both imaging and pathologic) are required to confirm the validity of this biomarker. Fur-thermore, while the methodology for assessing stromal TILs is standardized [22], inter-reader variability is a challenge [42], and variability in surgical versus biopsy specimens can be inconsistent. The lack of the functional details of the TILs in anti- or pro-tumor im-munity is also a challenge when using TILs as the standard for immune response. We worked to mitigate some of these shortcomings by having two academic breast pathologists read each slide independently and by scoring by deciles rather than an exact percentage. This limitation will need to be addressed in future studies. We augmented the assessment of immune response by also performing RNA sequencing to generate immune signature scores, which provide a more comprehensive answer on the overall anti- or pro-tumor status of the immune response. Although we did not co-localize the tissue and im-aging features in this study, this could be incorporated in future work. Finally, the short time frame between trastuzumab dose and post-treatment MRI (median of 14 days) in our study might not be sufficient to capture all immune responses, and stronger associations between imaging and TILs may be appreciated using a later post-treatment MRI.

## 5. Conclusions

In the future, imaging biomarker strategies may critically enhance clinicians’ ability to predict a tumor’s treatment response. The results of this pilot study demonstrate a correlation of MRI features with tumor immune microenvironment changes in early HER2+ breast cancer. Measures of DPE and pre-treatment ADC had the strongest correlation with immune response as defined by TILs and immune-active gene signature scores. Moreover, these data support the value of looking at both pre- and post-treatment MRI features to evaluate a change with treatment rather than assessing the pre-treatment MRI features alone. MRI measures hold potential to serve as biomarkers of tumor microenvironment alterations to non-invasively guide therapy selection in early breast cancer.

## Figures and Tables

**Table 1:** Patient Demographics and Tumor Grade.

Patient ID	Age at Diagnosis (Years)	ER Status (Allred Score)	Combined Histologic Grade	Days to Post-Trastuzumab MRI
1	41	Negative	2	16
2	39	Negative	3	11
3	37	Negative	3	14
4	57	Negative	2	14
5	40	Negative	3	18
6 *	60	Negative	1	14
7	61	Negative	3	11
8 *	53	Positive (3/8)	2	21
9	47	Positive (4/8)	3	15
10	37	Positive (7/8)	3	28
11	64	Positive (8/8)	2	14
12	69	Positive (8/8)	3	36
13 *^,+^	51	Positive (8/8)	3	14
14	52	Positive (8/8)	2	14

A total of 14 patients were enrolled in the study. Median patient age was 51 years old (37–69 range). Estrogen receptor status and Nottingham combined histologic grade are reported from the initial biopsies; median combined histologic grade was 3. The median number of days to post-trastuzumab MRI was 14 days (range 11–36). * received neoadjuvant chemotherapy and research biopsy; ^+^ received pertuzumab and trastuzumab. ER = estrogen receptor.

**Table 2:** Spearman Correlation Coefficients (ρ) of MRI Features with Tumor-Infiltrating Lymphocytes.

	Pre-Trastuzumab	Post-Trastuzumab	ΔTILs (ρ)
	TILs (ρ)	TILs (ρ)	
Functional Tumor Volume (FTV)			
FTV MRI #1	0.14	0.03	0.03
FTV MRI #2	−0.32	−0.19	−0.05
ΔFTV	−0.37	−0.18	0.19
Wash-Out Fraction (WF)			
WF MRI #1	0.34	0.22	0.51
WF MRI #2	0.08	0.18	0.26
ΔWF	−0.11	0.07	−0.11
Signal Enhancement Ratio (SER)			
SER MRI #1	0.28	0.22	0.33
SER MRI #2	0.23	0.36	0.38
ΔSER	0.25	0.35	0.32
Peak Percent Enhancement (PE)			
PE MRI #1	0.24	0.25	0.48
PE MRI #2	−0.1	-0.1	0.11
ΔPE	−0.45	-0.51	−0.69
Apparent Diffusion Coefficient (ADC)			
ADC MRI #1	−0.28	-0.51	−0.67
ADC MRI #2	−0.06	-0.27	−0.49
ΔADC	0.06	0.11	0.15

Functional tumor volume (FTV), combined wash-out volume percentage (WF), signal enhancement ratio (SER), and initial peak percent enhancement (PE) from the pre-treatment (MRI #1) and post-treatment (MRI #2) were correlated with the TILs in the pre- and post-treatment tissue specimens. For these calculations, 13/14 patients had complete datasets with paired MRIs and tissue samples available for correlation.

## Data Availability

Imaging, pathology and clinical data from this study may be made available upon request to the corresponding author.

## Supplementary Materials

The following supporting information can be downloaded at: https://www.mdpi.com/article/10.3390/cancers15174337/s1, Figure S1: Tumor-infiltrating lymphocyte (TIL) distribution by hormone receptor status before and after trastuzumab treatment. (A) Pre- and post-treatment TIL levels for patients with ER- breast cancer only. Post-treatment TILs were significantly higher than pre-treatment TILs (paired *t*-test, *p* < 0.05). (B) Pre- and post-treatment TIL levels for patients with ER+ breast cancer only. One ER+ patient had pre-treatment tissue available only due to lack of research biopsy after a single dose of trastuzumab. Post-treatment TILs trended towards being higher than pre-treatment TILs (*p* = 0.08); Figure S2: MRI features in groups with or without immune response. Immune response was defined as an increase in TIL content by more than a decile. (A) Peak percent enhancement. Significant difference observed between Pre-Tx and Post-Tx within the immune response cohort (paired *t*-test, *p* = 0.02). (B) Functional tumor volume (FTV), (C) signal enhancement ratio (SER), (D) combined wash-out volume (WF), and (E) apparent diffusion coefficient (ADC). Asterisk (*) indicates *p* < 0.05. Table S1: Individual patient data for imaging feature correlation with stromal tumor-infiltrating lymphocytes (sTILs). The stromal TILs are reported as deciles. Decile 1.0 = 0–9%, 2.0 = 10–19%, 3.0 = 20–29%, 4.0 = 30–39%, 5.0 = 40–49%, 6.0 = 50–59%, 7.0 = 60–69%, 8.0 = 70–79%, 9.0 = 80–89%, 10 = 90–99%. The functional tumor volume (FTV), combined wash-out volume percentage (WF), peak percent enhancement (PE), and signal enhancement ratio (SER) were all derived from the dynamic-contrast enhanced images. The apparent diffusion coefficient (ADC) was derived from a representative tumor plane from the diffusion-weighted imaging. Patient 8 did not have a post-trastuzumab tissue sample available for assessment.
