# Supplementary material for: Associations of Multiparametric Breast MRI Features, Tumor-Infiltrating Lymphocytes, and Immune Gene Signature Scores Following a Single Dose of Trastuzumab in HER2-Positive Early-Stage Breast Cancer
Source: Cancers (Basel). 2023 Aug 30;15(17):4337. doi: 10.3390/cancers15174337 (PMC10486523; doi:10.3390/cancers15174337)
Supplement: Supplementary file 1 [file cancers-15-04337-s001.zip › cancers-2573762-supplementary.pdf]

SUPPLEMENTAL FIGURES

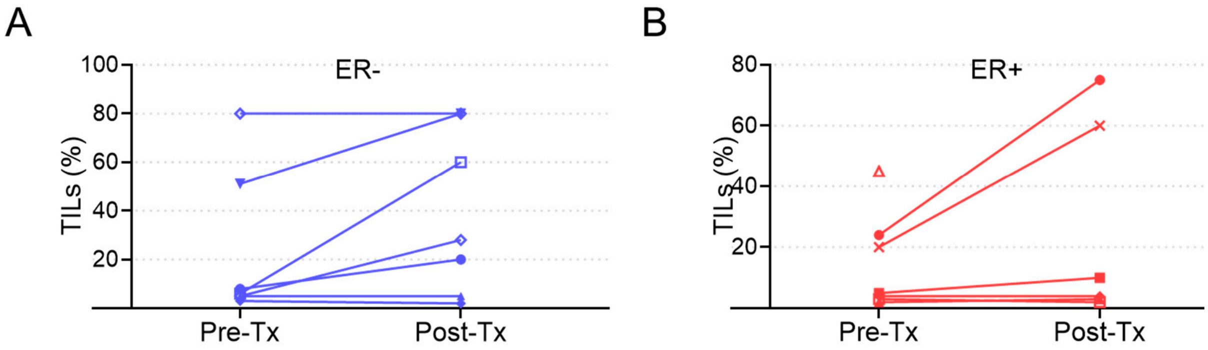

Figure S1. Tumor-infiltrating lymphocyte (TIL) distribution by hormone receptor status before and after trastuzumab treatment. A. Pre- and post-treatment TIL levels for patients with ER- breast cancer only. Post-treatment TILs were significantly higher than pre-treatment TILs (paired t-test, $p < 0.05$). **B** Pre- and post-treatment TIL levels for patients with ER+ breast cancer only. One ER+ patient had pre-treatment tissue available only due to lack of research biopsy after a single dose of trastuzumab. Post-treatment TILs trended towards being higher than pre-treatment TILs ($p = 0.08$).

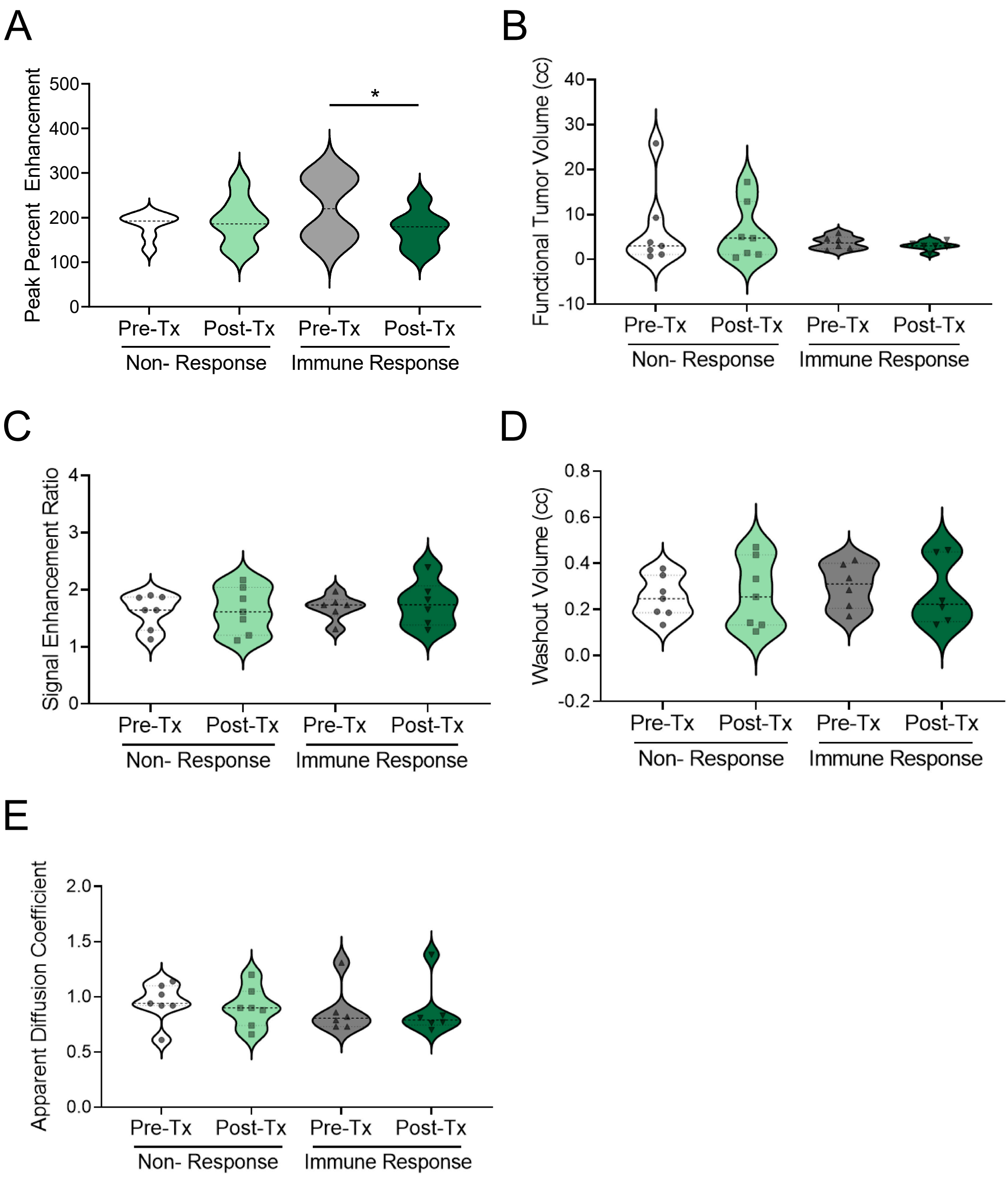

Figure S2. MRI features in groups with or without immune response. Immune response was defined as an increase in TIL content by more than a decile. **A** Peak percent enhancement. Significant difference observed between Pre-Tx and Post-Tx within the immune response cohort (paired t-test, $p = 0.02$). **B.** Functional tumor volume (FTV), **C.** signal enhancement ratio (SER), **D.** combined wash-out volume (WF), and **E)** apparent diffusion coefficient (ADC). Asterisk (*) indicates $p < 0.05$.

Table S1. Individual patient data for imaging feature correlation with stromal tumor-infiltrating lymphocytes (sTILs). The stromal TILs are reported as deciles. Decile 1.0 = 0-9%, 2.0 = 10-19%, 3.0 = 20-29%, 4.0 = 30-39%, 5.0 = 40-49%, 6.0 = 50-59%, 7.0 = 60-69%, 8.0 = 70-79%, 9.0 = 80-89%, 10 = 90-99%. The functional tumor volume (FTV), combined wash-out volume percentage (WF), peak percent enhancement (PE), and signal enhancement ratio (SER) were all derived from the dynamic-contrast enhanced images. The apparent diffusion coefficient (ADC) was derived from a representative tumor plane from the diffusion-weighted imaging. Patient 8 did not have a post-trastuzumab tissue sample available for assessment.

Pt ID	sTILs (decile)		fTV (cc)		WF		PE (%)		SER		ADC (x10^{-3} mm^2/sec)	
	Pre	Post	Pre	Post	Pre	Post	Pre	Post	Pre	Post	Pre	Post
**1**	1	3.5	4.3	4.3	0.17	0.237	173	139	1.62	1.65	0.82	0.81
**2**	6	9	4.6	3	0.285	0.132	137	118	1.73	1.41	0.86	0.83
**3**	9	9	3	1.4	0.185	0.254	192	185	1.64	1.84	0.92	0.9
**4**	1.5	7	3	2.7	0.336	0.208	303	190	1.73	1.82	0.73	0.7
**5**	1.5	3	2.5	1.2	0.216	0.151	163	168	1.31	1.29	1.31	1.38
**6**	1	1	25.8	12.9	0.132	0.142	201	186	1.64	1.48	1.14	1.2
**7**	1	1	9.3	17.2	0.277	0.332	201	279	1.86	2.04	1.1	1.05
**8**	5.5		19.3	1.5	0.554	0.254	210	111	1.97	1.16	0.78	1.03
**9**	3	8.5	2	3.5	0.395	0.456	291	242	1.97	2.39	0.73	0.77
**10**	3	7	5.9	3	0.414	0.447	267	191	1.78	1.96	0.79	0.76
**11**	1	1	0.7	0.4	0.246	0.132	128	136	1.13	1.11	0.92	0.9
**12**	1	1	1.1	1.1	0.377	0.437	191	198	1.9	1.61	0.94	0.88
**13**	1	2	2.1	5	0.348	0.47	207	231	1.87	2.17	0.61	0.66
**14**	1	1	3.8	4.7	0.19	0.104	160	124	1.29	1.2	1.02	0.74
**Median**	1.5	2.5	3	3	0.285	0.254	201	186	1.73	1.61	0.92	0.88
